# Cutaneous Adverse Drug Reactions (CADRs)—Statistical Analysis of the Causal Relationship between the Drug, Comorbidities, Cofactors, and the Cutaneous Reaction—A Single-Centered Study

**DOI:** 10.3390/ijerph19137982

**Published:** 2022-06-29

**Authors:** Natalia Machoń, Julia Lewandowska, Natalia Zdanowska, Waldemar Placek, Agnieszka Owczarczyk-Saczonek

**Affiliations:** 1 Medical Faculty, University of Warmia and Mazury in Olsztyn, 10-719 Olsztyn, Poland; julia.lewandowska.1@student.uwm.edu.pl; 2 Department of Dermatology, Sexually Transmitted Diseases and Clinical Immunology, University of Warmia and Mazury in Olsztyn, 10-229 Olsztyn, Poland; natalia.zdanowska@uwm.edu.pl (N.Z.); waldemar.placek@uwm.edu.pl (W.P.); agnieszka.owczarczyk@uwm.edu.pl (A.O.-S.)

**Keywords:** cutaneous manifestation, drug hypersensitivity, cutaneous adverse drug reactions (CADRs), suspected drugs

## Abstract

Cutaneous adverse drug reactions (CADRs) are among the most common types of drug hypersensitivity reactions. The purpose of this study was to evaluate the clinical spectrum of CADRs and to determine the causal relationship between drugs, comorbidities, cofactors or concomitant symptoms, and cutaneous reactions. A retrospective hospital-based study was carried out over a period of 10 years at the Department of Dermatology, Sexually Transmitted Diseases and Clinical Immunology at the University of Warmia and Mazury in Olsztyn to record various CADRs, comorbidities, cofactors, and the suspected drug in hospitalized patients. The data were subjected to statistical analysis. CADRs were diagnosed in a total of 140 patients, 32.14% of whom were men and 67.86% of whom were women. The mean age was 66.33 years. The most commonly suspected drugs were Allopurinol 12.86%, Amoxicillin with clavulanic acid 10%, Amoxicillin 9.29%, Paracetamol 6.43%, Metronidazole 5%, and Carbamazepine 5%. Attention should be paid to the possibility of using a substitute for a suspected drug if CADRs arise, or discontinuing a drug that is unjustifiably overused. The results of the present study should also prompt research into a potential treatment that could be implemented concurrently with a drug that has a high predisposition to cause CADRs.

## 1. Introduction

According to the World Health Organization, an adverse drug reaction is “a response to a drug, which is noxious and unintended, and which occurs at doses normally used in man for the prophylaxis, diagnosis, or therapy of disease, or for the modifications of physiological function”. The majority of such reactions involve the skin [1]. We may distinguish ten main types of cutaneous adverse drug reactions (CADRs): maculopapular rash, drug-induced hypersensitivity syndrome (DIHS), drug-induced urticaria, erythema dyschromicum perstans, erythema multiforme, acute generalized exanthematous pustulosis (AGEP), post-drug phototoxic and photoallergic reactions, symmetrical drug-related intertriginous and flexural exanthema, Stevens-Johnson syndrome (SJS)/toxic epidermal necrolysis (TEN), and drug-induced vasculitis [2]. A summary of the main features of these CADRs is presented in Table 1.

Maculopapular rash, the most common CADR, develops between 4 and 14 days after introducing the provocative drug. However, after re-taking the drug, the rash may appear within 1 to 2 days. Initially, erythematous patches or papules appear on the trunk and then spread symmetrically to the extremities. They may generalize and develop into erythroderma. Mucous membranes are usually unaffected. This cutaneous manifestation may be accompanied by pruritus and fever [3].

DIHS is a drug reaction with eosinophilia and systemic symptoms (DRESS). These acronyms are used interchangeably. This is due to the fact that, initially, the term drug hypersensitivity syndrome (DHS) was used, which summarized severe drug reactions. Subsequently, an entity was distinguished and named DRESS in Europe and renamed DIHS (drug-induced hypersensitivity syndrome) in Japan [1]. In addition, appropriate criteria for both DIHS and DRESS were established by the Japanese Research Committee on Severe Cutaneous Adverse Reactions and the RegiSCAR group, which differ slightly. For DIHS, one of the diagnostic criteria is the presence of HHV-6 reactivation, which is not present in DRESS. Therefore, it was concluded that the typical course of DIHS may correspond with a severe form of DRESS [4]. The most characteristic patterns of this reaction are: variable skin lesions, multiple organ involvement, lymphocyte activation, eosinophilia, and common viral reactivation [1]. The primary lesions include variable macules, papules, small superficial pustules or vesicles, eczema-like and target-like lesions, and purpura. Initially, the lesions develop on the upper parts of the trunk, face, and gradually involve the limbs, but they may also lead to the development of erythroderma [3].

Drug-induced urticaria is defined as round areas of raised erythema and swelling of the superficial dermis. The area of its occurrence is arbitrary and usually disappears without a trace within a few hours. Acute urticaria (less than 6 weeks) is usually triggered by an allergic reaction (IgE- or immune complex-dependent reactions) or a non-immune mechanism. The reaction occurs within hours or days after drug administration and resolves within a few days after withdrawal. However, reactions to drugs that are immunologically mediated require a period of sensitization. Usually, the initial course of drug therapy is quiescent. Nevertheless, an immune-mediated reaction may occur during the first exposure, but always after several days of treatment. It is because allergic urticaria is the cutaneous manifestation of a type I reaction. After a latent period of drug therapy, an IgE-mediated reaction may occur within minutes to an hour [5].

Erythema dyschromicum perstans is characterized by the presence of hyperpigmented patches of various sizes that localize on the face, trunk, and extremities. Initially, they appear as oval or round macular lesions with raised, erythematous borders. During the late stage, the spots turn grayish-blue and have unevenly demarcated borders [6].

The clinical picture of erythema multiforme initially includes numerous sharply demarcated red or pink macules that later become papular and gradually enlarge into plaques. The final lesion is round in shape and consists of 3 zones: a central dark red area, a lighter pink or edematous zone, and a peripheral red ring. Scabs or blisters sometimes appear in the center of the lesions. The lesions are symmetrically distributed with a tendency to occur on the distal parts of the extremities and then spread to the skin of the face, trunk, and proximal parts of the extremities, usually not exceeding 10% of the body surface. Mucosal involvement is minimal. Patients may experience itching and burning [7].

AGEP, characterized by the development of pustules on edematous erythema, primarily appear on the face and intertriginous area, but tend to disseminate within hours. The typical time interval between initial drug administration and the first onset of symptoms is 1–12 days. Other important findings are fever, leukocytosis, and neutrophilia. Moreover, transient renal failure may occur [3].

Post-drug phototoxic and photoallergic reactions result from exposure to a chemical agent (topical or systemic drug), and sunlight. Reactions are triggered by ultraviolet A (UVA) or ultraviolet B (UVB) radiation by an inflammatory response (phototoxicity) or a T-cell-mediated response (photoallergy). The classic eczematous rash of photoallergy and the typical exaggerated rash of phototoxicity are located on the face, neck, forearms, and hands, i.e., sun-exposed areas. Several other symptoms are possible, such as lichenoid eruptions, erythema multiforme, hyperpigmentation, and telangiectasias. Photoallergic reactions to drugs are less common than phototoxic ones and usually require minimal exposure to photosensitizing drugs and prior sensitization. The triggering mechanism is an immune-mediated type IV hypersensitivity reaction. They usually develop 24 h or more after the initial exposure and resemble eczematous dermatitis, which may spread beyond the sun-exposed skin. A phototoxic reaction results from direct damage to tissues and cells by a photoproduct. It is usually dose dependent and does not require prior sensitization. It occurs within minutes to hours after exposure to sunlight and clinically manifests as an exaggerated sunburn accompanied by burning and itching sensations in sun-exposed areas [8].

SDRIFE distribution involves the flexural and intertriginous regions. The typical clinical manifestation includes sharply demarcated erythema of the perioral and periorbital region, as well as the axillae and other intertriginous folds, with occasionally occurring pustules. Patients do not usually tend to have systemic symptoms. The period between the intake of the suspected drug and the appearance of the lesions is up to 7 days [3].

SJS and TEN are the most severe drug reactions, involving the skin and mucous membranes. They are considered the same disease entity, only with different variant severity. Initially, small vesicles appear on purple patches, which are widespread and occur mainly on the trunk. The skin is then usually painful. These blistering lesions develop rapidly on both the skin and mucous membranes, causing skin detachment with a positive Nikolsky sign. Hemorrhagic erosions of mucous membranes and fever are also present. The typical interval between the first dose of the drug and the onset of SJS/TEN ranges from 4 days to 4 weeks [3].

Drug-induced vasculitis is palpable purpura, petechiae, and bullae that appear 7–21 days after starting drug intake and may lead to necrosis on the lower extremities. The most common cofactors are: fever, arthralgia, hematuria, or proteinuria. Furthermore, lymphadenopathy and a serum sickness reaction may be suspected [3].

The purpose of this study involves the retrospective evaluation of the clinical spectrum of cutaneous adverse drug reactions (CADRs) in patients over a period of 10 years and the determination of the causal relationship between drugs, comorbidities, and cofactors or concomitant symptoms, and the CADRs.

## 2. Materials and Methods

A retrospective hospital-based study was carried out at the Department of Dermatology, Sexually Transmitted Diseases and Clinical Immunology at the University of Warmia and Mazury in Olsztyn, Poland, over a period of 10 years (January 2012 to January 2022). The case records of inpatients were screened for age, gender, comorbidities, type of drug reaction, suspected drug, time since drug intake, cofactor or concomitant symptoms, medications taken on a regular basis, and treatment implemented. As regards statistical analysis, we included the age, gender, CADRs, comorbidities, cofactors, and the suspected drug. The case records with the diagnosis of CADR made by a dermatologist were selected and researchers categorized individual CADRs into the following categories: maculopapular rash, DIHS, drug-induced urticaria, erythema dyschromicum perstans, erythema multiforme, acute generalized AGEP, post-drug phototoxic and photoallergic reactions, symmetrical drug-related intertriginous and flexural exanthema, SJS/TEN, and drug-induced vasculitis. Only drugs which certainly or probably triggered the reactions were included in the analysis. The data collected were subjected to statistical analysis. Data were coded and entered using the statistical package Statistica (Statistica 13.1, StatSoft, Kraków, Poland). They were summarized using the mean, standard deviation, median, minimum, and maximum in quantitative analysis. The frequency (count) and relative frequency (percentage) were used for categorical data. The χ2 test was performed to compare categorical data. The statistical results were based on Pearson′s Chi-square test. The level of statistical significance was set for values below 0.05 (*p* < 0.05).

## 3. Results

CADRs were diagnosed in a total of 140 patients, 45 (32.14%) of whom were men and 95 (67.86%) were women. The mean age of the patients was 66.33 years (minimal 12 years, maximal 96 years). The majority of the patients were in the age group of 70 years. The characteristics of the study population is presented in Table 2. Only SJS was significantly more common in men than in women (*p* = 0.01096). Overall, no statistical significance was observed with respect to age regarding the predisposition to CADRs, or between genders regarding the clinical data.

The observed CADRs included MPR 23.57%, DIHS 37.14%, drug-induced urticaria 12.86%, erythema dyschromicum perstans 9.29%, erythema multiforme 5.00%, AGEP 2.86%, post-drug phototoxic and photoallergic reactions 2.86%, SDRIFE 2.86%, SJS 2.14%, and drug-induced vasculitis 1.43%. These results are presented in Table 3.

The most commonly suspected drugs were: Amoxicillin with clavulanic acid 10%, Amoxicillin 9.29%, Paracetamol 6.43%, Metronidazole 5%, and Carbamazepine 5%. Both Amoxicillin with clavulanic acid 10% and Amoxicillin 9.29% were suspected of causing CADRs and the percentage difference between them was statistically insignificant, indicating the influence of the antibiotic rather than the β-lactamase inhibitor as the causative agent of the rashes. A summary of statistically significant factors influencing the emergence of CADRs is presented in Table 4 and Figure S1.

### 3.1. Maculopapular Rash

No statistically significant comorbidities were found in the occurrence of maculopapular rash. However, the most common concomitant symptoms were eosinophilia (*p* = 0.04423) and elevated liver parameters (*p* = 0.06677). The predominant drugs inducing its occurrence were: Amoxicillin (*p* = 0.04400), Metronidazole (*p* = 0.03179), Allopurinol (*p* = 0.05372), and Acetylcysteine (*p* = 0.01032).

### 3.2. DIHS

Statistically significant comorbidities among patients who developed DIHS included chronic heart failure (CHF) (*p* = 0.01094), chronic obstructive pulmonary disease (COPD) or asthma (*p* = 0.02564). An increased C-reactive protein (CRP) or accelerated erythrocyte sedimentation rate (ESR) (*p* = 0.03549) were cofactors affecting the development of CADR, while leukocytosis (*p* = 0.04404), eosinophilia (*p* = 0.00000), elevated liver parameters (*p* = 0.04841), and impaired renal parameters (*p* = 0.00012) were concomitant symptoms with high statistical significance. Ketoprofen (*p* = 0.04341) and Allopurinol (*p* = 0.00097) were drugs that predominantly induced this CADR.

### 3.3. Drug-Induced Urticaria

Nicotinism (*p* = 0.00898) and hypercholesterolemia (*p* = 0.03199) were statistically significant comorbidities in the occurrence of drug-induced urticaria. Fever was one of the major and statistically significant cofactors in the development of this rash (*p* = 0.01498). Suspected drugs, provoking the occurrence of this CADR, mainly included: platelet-rich plasma (*p* = 0.00898), Mebeverine (*p* = 0.00898), Omeprazole (*p* = 0.00021), Celecoxib (*p* = 0.00898), Potassium chloride (*p* = 0.00898), Pantoprazole (*p* = 0.00898), Enoxaparin (*p* = 0.00898), Clemastine (*p* = 0.00898), and Hesperidin (*p* = 0.00898).

### 3.4. Erythema Dyschromicum Perstans

The concomitant diseases that were important in the development of erythema dyschromicum perstans included osteoporosis (*p* = 0.01596) and acute renal failure (*p* = 0.00171). No high statistical significance was observed as regards the cofactors or concomitant symptoms. The drugs suspected of provoking this CADR were Diclofenac (*p* = 0.01596), Drotaverine (*p* = 0.04569), Naphazoline (*p* = 0.00171), Sulfathiazole (*p* = 0.00171), Xylometazoline (*p* = 0.00171), herbal teas (*p* = 0.00171), Cetirizine (*p* = 0.00171), Inosine pranobex (*p* = 0.00171), Tolperisone (*p* = 0.04569), and Simvastatin (*p* = 0.00171).

### 3.5. Erythema Multiforme

Spiramycin (*p* = 0.01326) was the only drug that showed a significant correlation with the occurrence of erythema multiforme. Comorbidities typically accompanying that rash were rheumatoid arthritis (*p* = 0.00327) and Alzheimer’s disease (*p* = 0.00327). Based on the patients’ medical histories, no statistically significant cofactors or concomitant symptoms were noted.

### 3.6. AGEP

The medical history of AGEP patients included comorbidities with statistical significance such as psychiatric illness (*p* = 0.01912), atrial fibrillation (*p* = 0.00442), and neoplastic disease (*p* = 0.03795). Leukocytosis (*p* = 0.03090) was the only statistically significant concomitant symptom in case of this rash. The drugs causing AGEP were Bisoprolol (*p* = 0.00006), ASA (*p* = 0.00000), CT contrast (*p* = 0.01912), Ascorbic Acid (*p* = 0.00136), Pheniramine (*p* = 0.00136), Rutoside (*p* = 0.00000), Salicylamide (*p* = 0.00000), Leuprorelin (*p* = 0.00000), and Flutamide (*p* = 0.00000).

### 3.7. Post-Drug Phototoxic and Photoallergic Reactions

Post-drug phototoxic and photoallergic reactions were characterized by UV exposure (*p* = 0.0000). Statistical significance was observed in case of Naproxen (*p* = 0.00136), Ibuprofen (*p* = 0.00136), Penicillamine (*p* = 0.00000), and Tolperisone (*p* = 0.00006). Based on patients’ medical histories, there were no statistically significant comorbidities.

### 3.8. SDRIFE

SDRIFE patients were characterized by the presence of anal varices (*p* = 0.00136), chronic dermatological disease (*p* = 0.01868) or cancer (*p* = 0.03795) in their medical history. As regards SDRIFE, statistical significance occurred for Metronidazole (*p* = 0.06259), chemotherapy (*p* = 0.00006), Dextromethorphan (*p* = 0.00000), Pseudoephedrine (*p* = 0.00000), and Bromazepam (*p* = 0.00000). No high statistical significance was observed with regard to cofactors or concomitant symptoms.

### 3.9. SJS

The occurrence of SJS was significantly higher among individuals with a history of epilepsy (*p* = 0.01203). Statistical significance was noted for the following drugs provoking the appearance of this rash: Carbamazepine (*p* = 0.02283) and Rituximab (*p* = 0.00000). No high statistical significance occurred for cofactors or concomitant symptoms.

### 3.10. Drug-Induced Vasculitis

Only one drug (Ibrutinib) demonstrated statistical significance (*p* = 0.00000) in the induction of drug-induced vasculitis. Neither significant comorbidities nor cofactors or concomitant symptoms were identified for this type of CARD.

## 4. Discussion

The investigation for CADR confirmation involves the analysis of the time from the initial exposure or re-exposure to the appearance of the skin lesion, resolution of lesions after the discontinuation of the drug, nature of lesions, and a history of similar reactions to the suspected drug.

The study revealed that women were more prone to develop CADR, which was also proved by other studies [9]. However, this predisposition may be due to the fact that women were more likely to consult a doctor after noting a lesion than men [10]. Regarding the age, the majority of the patients were around 70 years old. The median age of the patients that presented CADRs was slightly higher in comparison with previous studies, i.e., SJS and TEN usually appeared at the age of 53.4 years, AGEP at 56 years, and DRESS at 41.5 years for women, and 57 years for men [1].

Based on our statistics, DIHS and maculopapular rash followed by drug-induced urticaria were the most frequently observed CADRs in the study. However, in previous studies, maculopapular rash (46.3%) was by far the most common CADR, followed by urticaria (14.2%), and pruritus (12.7%) [11].

The medications that most commonly caused skin reactions were: Allopurinol which induced both DIHS (25.00%) and maculopapular rash (3.03%), Amoxicillin that induced maculopapular rash (18.18%), Metronidazole provoking either maculopapular rash (12.12%) or SDRIFE (25.00%), and Carbamazepine inducing SJS (33.33%). Referring to another study by Chopra et al., the most common drugs that caused skin rashes were antibiotics, followed by non-steroidal anti-inflammatory drugs (NSAIDs) [11]. Our results concerning antibiotics, namely Amoxicillin, were similar. However, our study did not reveal such a high rash rate occurring after NSAIDs. To summarize the statistics, it may be concluded that, depending on the drug administered, a different cutaneous manifestation occurred in almost every case. The exceptions that appeared to be statistically significant and induced more than one CADR were: Allopurinol, which induced both maculopapular rash (3.03%) and DIHS (25.00%), Metronidazole, which induced maculopapular rash (12.12%) and SDRIFE, and Tolperisone, which induced either erythema perstans (7.69%) or post-drug phototoxic and photoallergic reactions (25.00%). The high incidence of CADRs being induced by those drugs may be related to the more frequent prescription. It is worth noting Allopurinol, which is frequently prescribed to decrease high blood uric acid levels. An increased risk of CADRs, and even mortality, were reported in people treated with this drug for asymptomatic hyperuricemia. Therefore, it should be emphasized that current indications for its use are: gout, recurrent gout (defined as ≥2 per year), chronic kidney disease ≥ stage 2, or urolithiasis. The routine use of any urate-lowering therapy for asymptomatic hyperuricemia is not recommended, because of its frequent side effects. In addition, other alternative urate-lowering treatments may be used if patients with gout develop a CADR after using Allopurinol. The dechallenge of the offending drug was carried out in all cases immediately after the identification of the CADR and treated appropriately. In most cases, it resulted in a rapid resolution of lesions [12,13].

As regards concomitant symptoms, they occurred significantly more often, according to the characteristics of individual CADRs. We concluded that elevated liver parameters and eosinophilia were the most commonly associated with the occurrence of maculopapular rash and DIHS. Moreover, elevated CRP or accelerated ESR were typically present as cofactors in DIHS, while impaired renal parameters acted as an accompanying symptom in this particular CADR. Fever was characteristic as a cofactor only for drug-induced urticaria. In contrast, UV exposure was an obvious cofactor in the induction of drug-induced phototoxic and photoallergic reactions.

The presence of comorbidities is unlikely to directly affect the induction of CADRs. Given the older age of most hospitalized patients, their association with skin reaction was mainly due to polypharmacy, which increased the risk of ADR occurrence due to drug interactions. Age-related changes in pharmacokinetics and pharmacodynamics, associated with deteriorating renal or hepatic function, were also related to a higher risk of developing ADRs [14].

One limitation of our study is that it is a retrospective and single-center study, so technical and selection biases are inevitable. Despite the lack of discrimination based on race, there was a predominance of the Polish population among the included patients. Another limitation is related to the fact that there are no universal rules to identify with certainty the drug that would cause CADRs and this information depended on the subjective examination of the patient. In addition, the assessment of skin lesions and their classification into different types of CADRs was subjective, conducted by a physician. The assessment of the time from drug intake to the appearance of skin lesions was also limited, as patients did not always remember this information. Moreover, the patients did not undergo long-term follow-up after hospital discharge, so long-term skin lesion evaluation was not always possible.

## 5. Conclusions

Many drugs prescribed on a large scale may contribute to the development of CADRs. Therefore, it is important to take this aspect into consideration and be cautious when prescribing them to patients. In particular, caution should be taken when prescribing routine drugs that are not the current standard of medical treatment. In the event of the occurrence of skin rashes, the use of a substitute drug or discontinuation of an inappropriately overused drug should be considered. In addition, it is worth checking the patient’s comorbidities, as some may be associated with an increased risk of developing a particular rash. The results of the present study should also prompt research into a potential treatment that could be implemented concurrently with a drug that has a high predisposition to cause CADRs.

## Figures and Tables

**Table 1:** Characteristics of CADRs.

	*CADRs*									
	*MPR*	*DIHS*	*DIU*	*EDP*	*EM*	*AGEP*	*PDPAPR*	*SDRIFE*	*SJS*	*DIV*
***Features***	-erythematous patches/papules -trunk, extremities -pruritus, fever	-various lesions -trunk, face, limbs -multiple organ involvement, eosinophilia	-round areas of raised erythema -swelling -arbitrary location	-hyperpigmented patches -face, trunk, extremities -initially: oval/round macular lesions with erythematous borders -late stage: grayish-blue spots with demarcated borders	-round lesions -3 zones: central dark red area, lighter pink zone, peripheral red ring -symmetrical: extremities, face, trunk -itching and burning	-pustules on edematous erythema -face and intertriginous area, dissemination -1–12 days -fever, leukocytosis, neutrophilia	-exposure to drug and UVA or UVB -eczematous rash or exaggerated rash -face, neck, forearms, hands, -24 h or more -no prior sensitization	-flexural and intertriginous regions -sharply demarcated erythema, pustules -perioral, periorbital, intertriginous folds -up to 7 days	-the most severe -skin mucous membranes -widespread small vesicles on purple patches -painful skin -Nikolsky sign -hemorrhagic erosions -fever -4 days to 4 weeks	-palpable purpura, petechiae, bullae -can lead to necrosis -7 to 21 days

Abbreviations: AGEP, acute generalized exanthematous pustulosis; DIHS, drug-induced hypersensitivity syndrome; DIU, drug-induced urticaria; DIV, drug-induced vasculitis; EDP, erythema dyschromicum perstans; EM, erythema multiforme; MPR, maculopapular rash; PDPAPR, post-drug phototoxic and photoallergic reactions; SDRIFE, symmetrical drug-related intertriginous and flexural exanthema; SJS, Stevens-Johnson syndrome.

**Table 2:** Characteristics of the study population—Number of people.

		*CADRs*									
		*MPR*	*DIHS*	*DIU*	*EDP*	*EM*	*AGEP*	*PDPAPR*	*SDRIFE*	*SJS*	*DIV*
***Demographics***	*<70 years old*	16	20	8	8	4	2	3	2	2	0
	*≥70 years old*	17	32	10	5	3	2	1	2	1	2
	*Women*	24	35	14	8	5	2	4	2	0	1
	*Men*	9	17	4	5	2	2	0	2	3	1

Abbreviations: AGEP, acute generalized exanthematous pustulosis; DIHS, drug-induced hypersensitivity syndrome; DIU, drug-induced urticaria; DIV, drug-induced vasculitis; EDP, erythema dyschromicum perstans; EM, erythema multiforme; MPR, maculopapular rash; PDPAPR, post-drug phototoxic and photoallergic reactions; SDRIFE, symmetrical drug-related intertriginous and flexural exanthema; SJS, Stevens-Johnson syndrome.

**Table 3:** The clinical spectrum of CADRs with percentages of patients.

	*CADRs*									
	*MPR*	*DIHS*	*DIU*	*EDP*	*EM*	*AGEP*	*PDPAPR*	*SDRIFE*	*SJS*	*DIV*
*Percentage of patients*	23.57%	37.14%	12.86%	9.29%	5.00%	2.86%	2.86%	2.86%	2.14%	1.43%

Abbreviations: AGEP, acute generalized exanthematous pustulosis; DIHS, drug-induced hypersensitivity syndrome; DIU, drug-induced urticaria; DIV, drug-induced vasculitis; EDP, erythema dyschromicum perstans; EM, erythema multiforme; MPR, maculopapular rash; PDPAPR, post-drug phototoxic and photoallergic reactions; SDRIFE, symmetrical drug-related intertriginous and flexural exanthema; SJS, Stevens-Johnson syndrome.

**Table 4:** Statistically significant factors influencing the emergence of CADRs.

		*Suspected Drugs*	*Cofactors or Concomitant Symptoms*	*Comorbidity*
*CADRs*	*MPR*	**Acetylcysteine** (*p* = 0.01032) **Allopurinol** (*p* = 0.05372) **Amoxicillin** (*p* = 0.04400) **Metronidazole** (*p* = 0.03179)	**Elevated liver parameters** (*p* = 0.06677) **Eosinophilia** (*p* = 0.04423)	-
	*DIHS*	**Allopurinol** (*p* = 0.00097) **Ketoprofen** (*p* = 0.04341)	**Increased CRP or accelerated ESR** (*p* = 0.03549) **Elevated liver parameters** (*p* = 0.04841) **Eosinophilia** (*p* = 0.00000) **Impaired renal parameters** (*p* = 0.00012) **Leukocytosis** (*p* = 0.04404)	**CHF** (*p* = 0.01094) **COPD or asthma** (*p* = 0.02564)
	*DIU*	**Celecoxib** (*p* = 0.00898) **Clemastine** (*p* = 0.00898) **Enoxaparin** (*p* = 0.00898) **Hesperidin** (*p* = 0.00898) **Mebeverine** (*p* = 0.00898) **Omeprazole** (*p* = 0.00021) **Pantoprazole** (*p* = 0.00898) **Platelet-rich plasma** (*p* = 0.00898) **Potassium chloride** (*p* = 0.00898)	**Fever** (*p* = 0.01498)	**Hypercholesterolemia** (*p* = 0.03199) **Nicotinism** (*p* = 0.00898)
	*EDP*	**Cetirizine** (*p* = 0.00171) **Diclofenac** (*p* = 0.01596) **Drotaverine** (*p* = 0.04569) **Herbal teas** (*p* = 0.00171) **Inosine pranobex** (*p* = 0.00171) **Naphazoline** (*p* = 0.00171) **Simvastatin** (*p* = 0.00171) **Sulfathiazole** (*p* = 0.00171) **Tolperisone** (*p* = 0.04569) **Xylometazoline** (*p* = 0.00171)	-	**Acute renal failure** (*p* = 0.00171) **Osteoporosis** (*p* = 0.01596)
	*EM*	**Spiramycin** (*p* = 0.01326)	-	**Alzheimer’s Disease** (*p* = 0.00327) **RA** (*p* = 0.00327)
	*AGEP*	**ASA** (*p* = 0.00000) **Ascorbic Acid** (*p* = 0.00136) **Bisoprolol** (*p* = 0.00006) **CT contrast** (*p* = 0.01912) **Flutamide** (*p* = 0.00000) **Leuprorelin** (*p* = 0.00000) **Pheniramine** (*p* = 0.00136) **Rutoside** (*p* = 0.00000) **Salicylamide** (*p* = 0.00000)	**Leukocytosis** (*p* = 0.03090)	**AF** (*p* = 0.00442) **Neoplastic disease** (*p* = 0.03795) **Psychiatric illness** (*p* = 0.01912)
	*PDPAPR*	**Ibuprofen** (*p* = 0.00136) **Naproxen** (*p* = 0.00136) **Penicillamine** (*p* = 0.00000) **Tolperisone** (*p* = 0.00006)	**UV exposure** (*p* = 0.0000)	-
	*SDRIFE*	**Bromazepam** (*p* = 0.00000) **Chemotherapy** (*p* = 0.00006) **Dextromethorphan** (*p* = 0.00000) **Metronidazole** (*p* = 0.06259) **Pseudoephedrine** (*p* = 0.00000)	-	**Anal varices** (*p* = 0.00136) **Cancer** (*p* = 0.03795) **Chronic dermatological disease** (*p* = 0.01868)
	*SJS*	**Carbamazepine** (*p* = 0.02283) **Rituximab** (*p* = 0.00000)	-	**Epilepsy** (*p* = 0.01203)
	*DIV*	**Ibrutinib** (*p* = 0.00000)	-	-

Abbreviations: AGEP, acute generalized exanthematous pustulosis; DIHS, drug-induced hypersensitivity syndrome; DIU, drug-induced urticaria; DIV, drug-induced vasculitis; EDP, erythema dyschromicum perstans; EM, erythema multiforme; MPR, maculopapular rash; PDPAPR, post-drug phototoxic and photoallergic reactions; SDRIFE, symmetrical drug-related intertriginous and flexural exanthema; SJS, Stevens-Johnson syndrome.

## Data Availability

Data sharing not applicable.

## Supplementary Materials

The following supporting information can be downloaded at: https://www.mdpi.com/article/10.3390/ijerph19137982/s1, Figure S1. Statistically significant drugs causing CADRs. (A) Most commonly suspected drugs; (B) Most commonly suspected drugs causing maculopapular rash; (C) Most commonly suspected drugs causing drug-induced hypersensitivity syndrome; (D) Most commonly suspected drugs causing drug-induced urticaria; (E) Most commonly suspected drugs causing erythema dyschromicum perstans; (F) Most commonly suspected drugs causing erythema multiforme; (G) Most commonly suspected drugs causing acute generalized exanthematous pustulosis; (H) Most commonly suspected drugs causing post-drug phototoxic and photoallergic reactions; (I) Most commonly suspected drugs causing symmetrical drug-related intertriginous and flexular exanthema; (J) Most commonly suspected drugs causing Stevens-Johnson syndrome; (K) Most commonly suspected drugs causing drug-induced vasculitis.
