# Supplementary material for: Cutaneous Adverse Drug Reactions (CADRs)—Statistical Analysis of the Causal Relationship between the Drug, Comorbidities, Cofactors, and the Cutaneous Reaction—A Single-Centered Study
Source: Int J Environ Res Public Health. 2022 Jun 29;19(13):7982. doi: 10.3390/ijerph19137982 (PMC9265797; doi:10.3390/ijerph19137982)
Supplement: Supplementary file 1 [file ijerph-19-07982-s001.zip › ijerph-1729966-supplementary.pdf]

Supplementary Materials

Cutaneous Adverse Drug Reactions (CADRs)—Statistical Analysis of the Causal Relationship between the Drug, Comorbidities, Cofactors, and the Cutaneous Reaction—A Single-Centered Study

Natalia Machoń [1,*], Julia Lewandowska [1], Natalia Zdanowska [2], Waldemar Placek [2] and Agnieszka Owczarczyk-Saczonek [2]

[1] Medical Faculty, University of Warmia and Mazury in Olsztyn, 10-719 Olsztyn, Poland; julia.lewandowska.1@student.uwm.edu.pl
[2] Department of Dermatology, Sexually Transmitted Diseases and Clinical Immunology, University of Warmia and Mazury in Olsztyn, 10-229 Olsztyn, Poland; natalia.zdanowska@uwm.edu.pl (N.Z.); waldemar.placek@uwm.edu.pl (W.P.); agnieszka.owczarczyk@uwm.edu.pl (A.O.-S.)
* Correspondence: mnatalia000@gmail.com

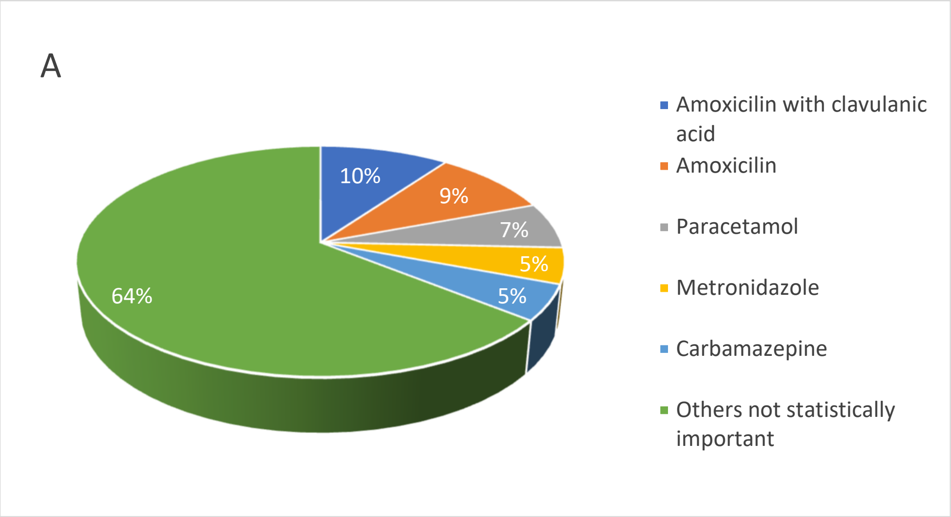

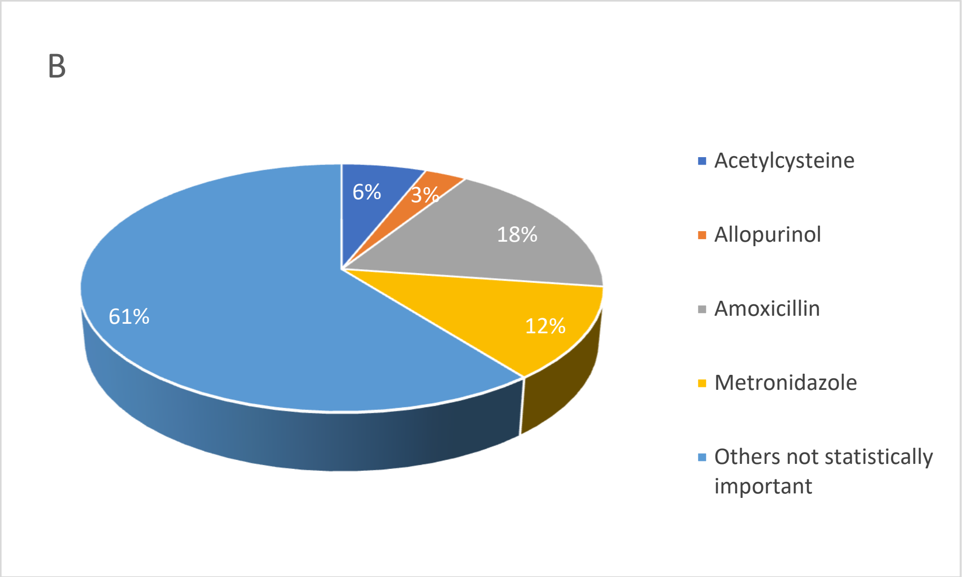
B
6%
3%
18%
12%
61%
Acetylcysteine
Allopurinol
Amoxicillin
Metronidazole
Others not statistically important

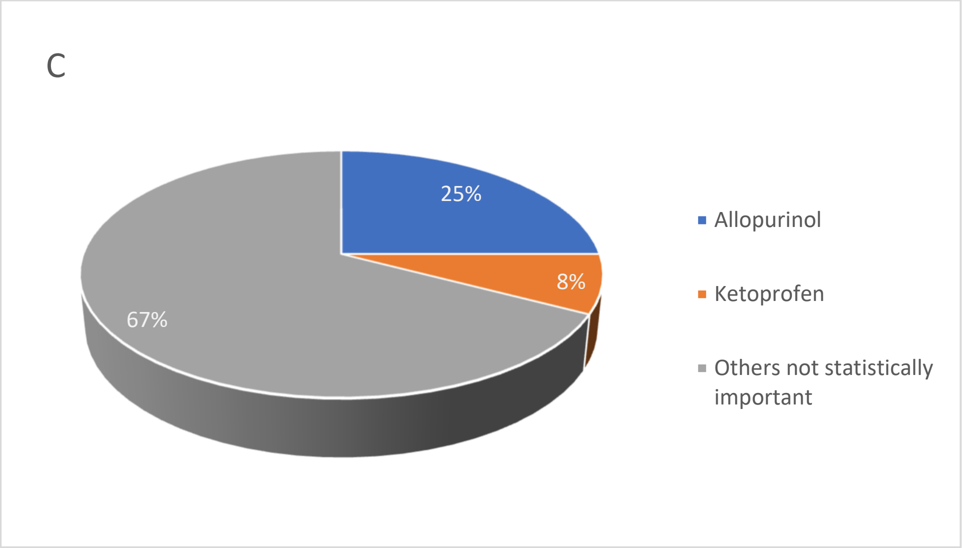
C
25%
8%
67%
Allopurinol
Ketoprofen
Others not statistically important

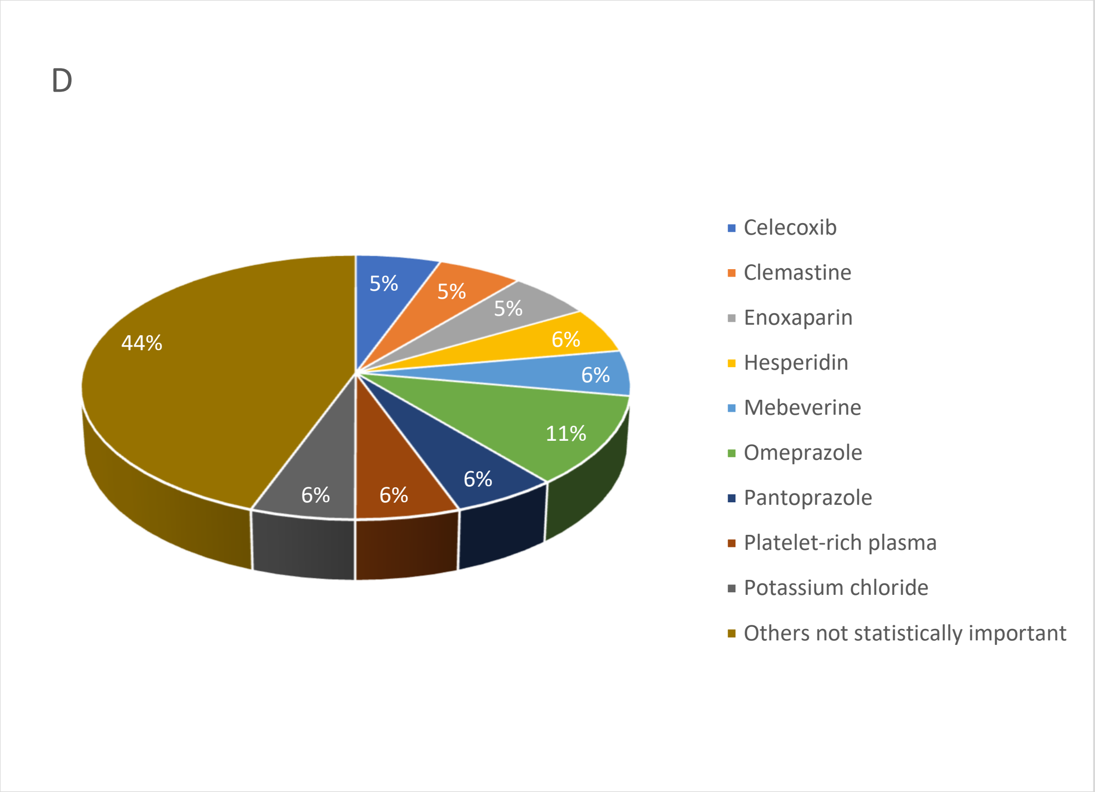
D
5%
5%
5%
6%
6%
11%
6%
6%
6%
44%
Celecoxib
Clemastine
Enoxaparin
Hesperidin
Mebeverine
Omeprazole
Pantoprazole
Platelet-rich plasma
Potassium chloride
Others not statistically important

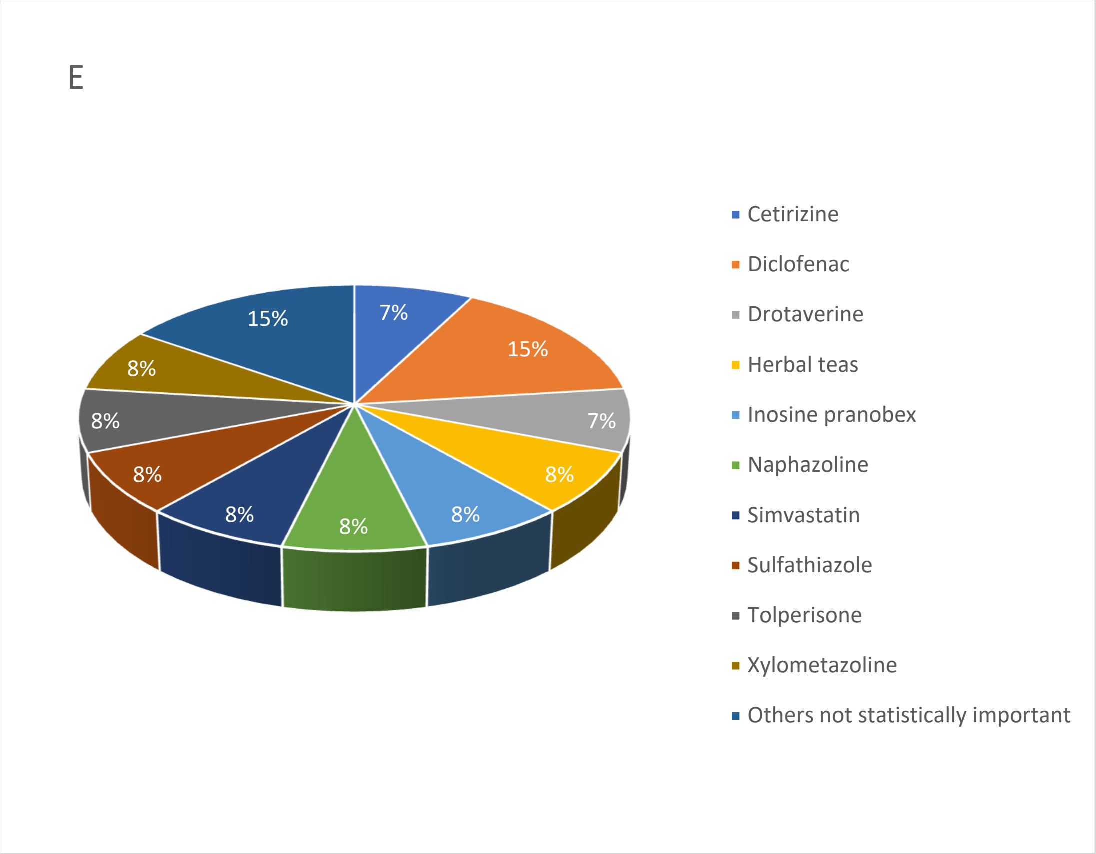
E
7%
15%
7%
8%
8%
8%
8%
8%
8%
8%
15%
Cetirizine
Diclofenac
Drotaverine
Herbal teas
Inosine pranobex
Naphazoline
Simvastatin
Sulfathiazole
Tolperisone
Xylometazoline
Others not statistically important

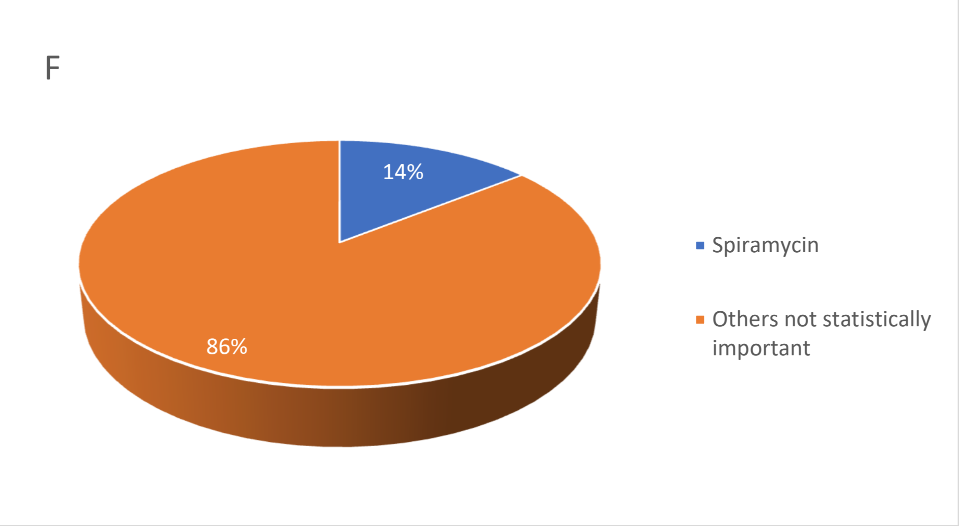
F
14%
86%
Spiramycin
Others not statistically important

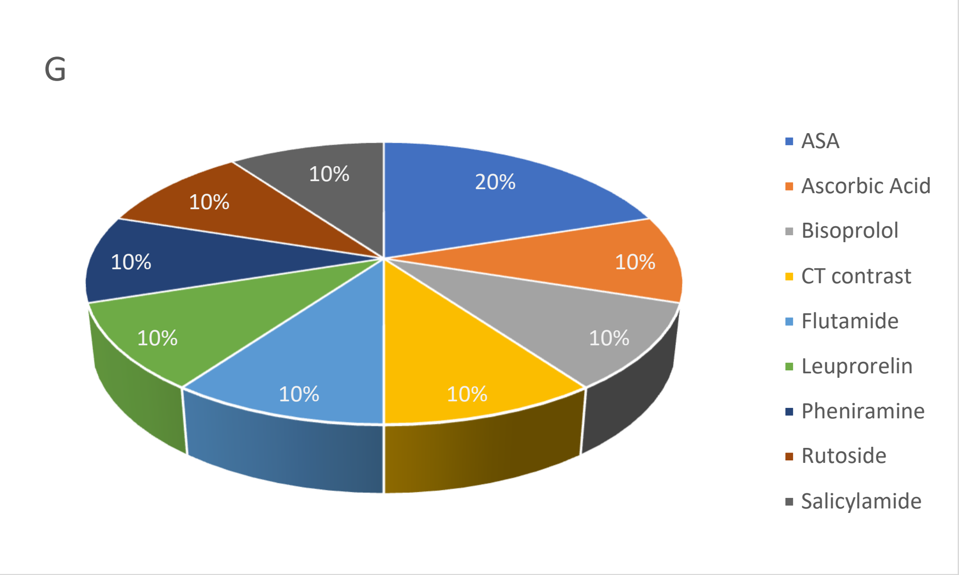
G
20%
10%
10%
10%
10%
10%
10%
10%
10%
ASA
Ascorbic Acid
Bisoprolol
CT contrast
Flutamide
Leuprorelin
Pheniramine
Rutoside
Salicylamide

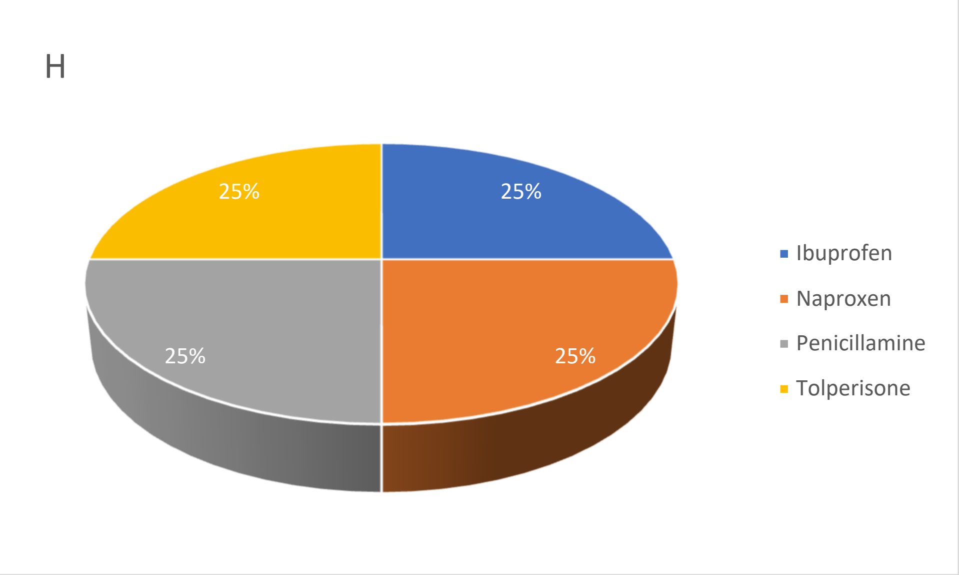
H
25%
25%
25%
25%
Ibuprofen
Naproxen
Penicillamine
Tolperisone

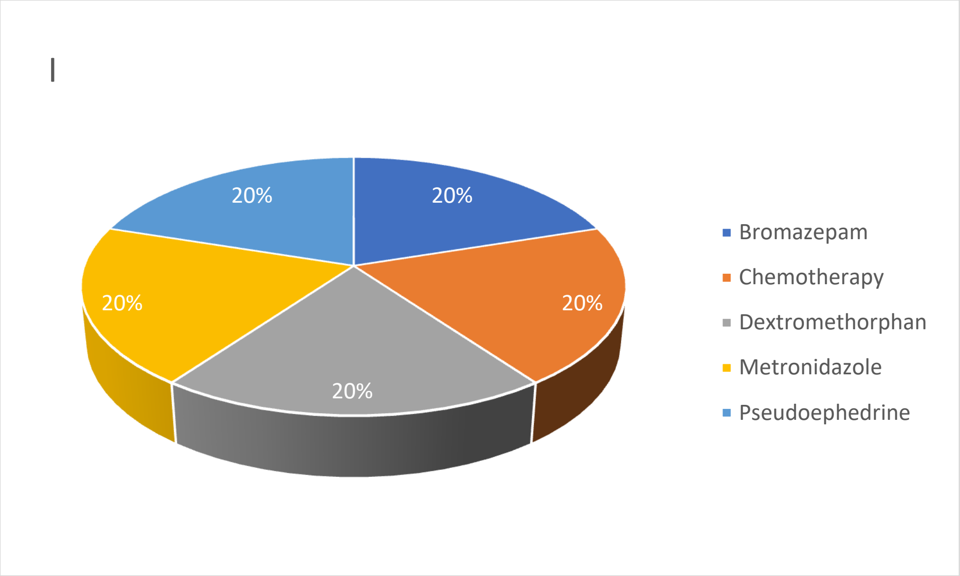

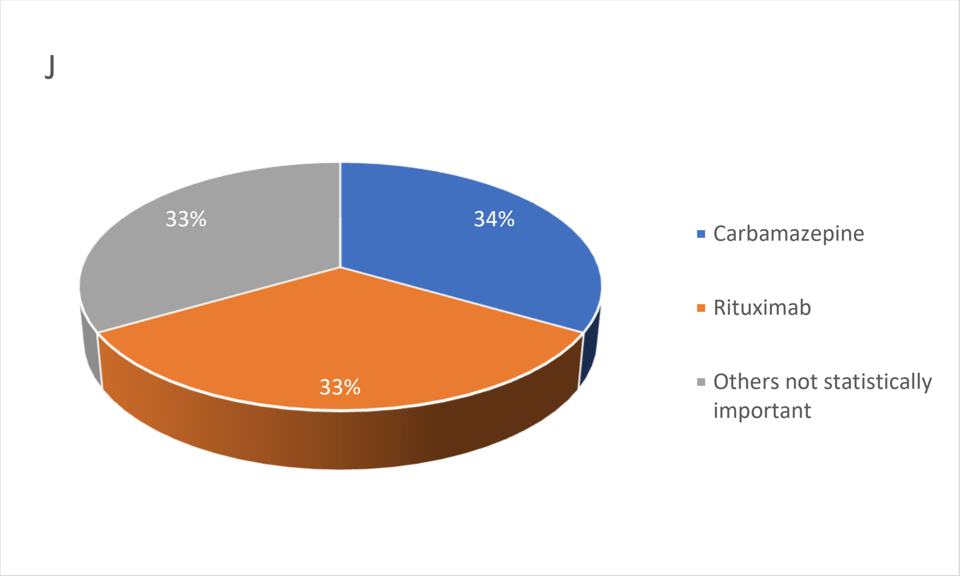

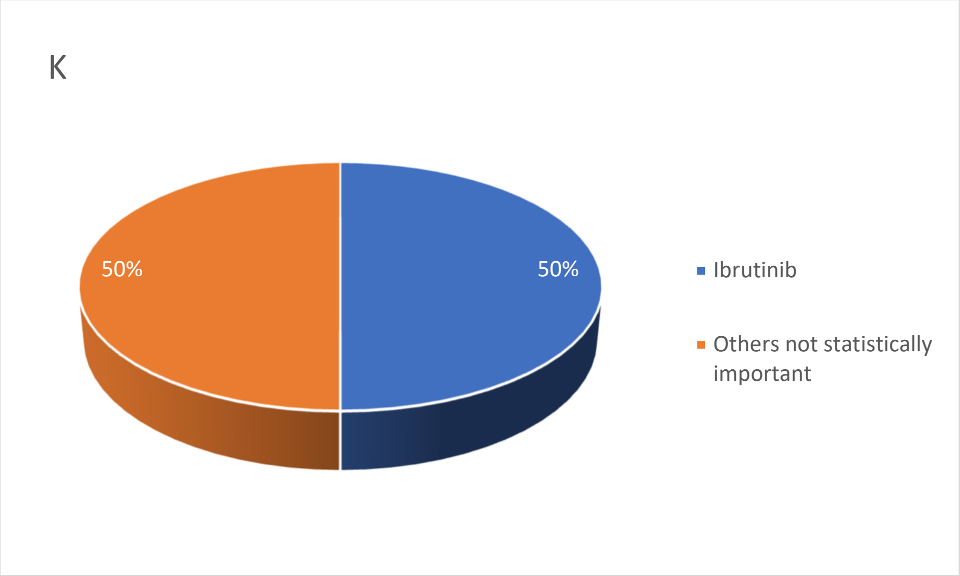

Figure S1. Statistically significant drugs causing CADRs. (A) Most common suspected drugs; (B) Most common suspected drugs causing maculopapular rash; (C) Most common suspected drugs causing drug-induced hypersensitivity syndrome; (D) Most common suspected drugs causing drug-induced urticaria; (E) Most common suspected drugs causing erythema dyschromicum perstans; (F) Most common suspected drugs causing erythema

multiforme; (G) Most common suspected drugs causing acute generalized exanthematous pustulosis; (H) Most common suspected drugs causing post-drug phototoxic and photoallergic reactions; (I) Most common suspected drugs causing symmetrical drug-related intertriginous and flexular exanthema; (J) Most common suspected drugs causing Stevens-Johnson syndrome; (K) Most common suspected drugs causing drug-induced vasculitis.
